# Effective combination treatment of GD2-expressing neuroblastoma and Ewing's sarcoma using anti-GD2 ch14.18/CHO antibody with Vγ9Vδ2+ γδT cells

**DOI:** 10.1080/2162402X.2015.1025194

**Published:** 2015-04-27

**Authors:** Jonathan P H Fisher, Barry Flutter, Florian Wesemann, Jennifer Frosch, Claudia Rossig, Kenth Gustafsson, John Anderson

**Affiliations:** 1 University College London Institute of Child Health; Developmental Biology and Cancer Section; London, UK; 2 Department of Pediatric Hematology and Oncology; University Children´s Hospital Muenster; Muenster, Germany; 3 University College London Institute of Child Health; Infection, Immunity, Inflammation and Physiological Medicine Section; London, UK

**Keywords:** antibody-dependent cytotoxicity, Ewing's Sarcoma, Gamma-delta T cells, neuroblastoma, translational

## Abstract

Gamma delta T lymphocytes (γδT cells) have pleiotropic properties including innate cytotoxicity, which make them attractive effectors for cancer immunotherapy. Combination treatment with zoledronic acid and IL-2 can activate and expand the most common subset of blood γδT, which express the Vγ9Vδ2 T cell receptor (TCR) (Vδ2 T cells). Vγ9Vδ2 T cells are equipped for antibody-dependent cell-mediated cytotoxicity (ADCC) through expression of the low-affinity FcγR CD16. GD2 is a highly ranked tumor associated antigen for immunotherapy due to bright expression on the cell surface, absent expression on normal tissues and availability of therapeutic antibodies with known efficacy in neuroblastoma. To explore the hypothesis that zoledronic acid, IL-2 and anti-GD2 antibodies will synergize in a therapeutic combination, we evaluated *in vitro* cytotoxicity and tumor growth inhibition in the GD2 expressing cancers neuroblastoma and Ewing's sarcoma. Vδ2 T cells exert ADCC against GD2-expressing Ewing's sarcoma and neuroblastoma cell lines, an effect which correlates with the brightness of GD2 expression. In an immunodeficient mouse model of small established GD2-expressing Ewing's sarcoma or neuroblastoma tumors, the combination of adoptively transferred Vδ2+ T cells, expanded *in vitro* with zoledronic acid and IL-2, with anti-GD2 antibody ch14.18/CHO, and with systemic zoledronic acid, significantly suppressed tumor growth compared to antibody or γδT cell-free controls. Combination treatment using ch14.18/CHO, zoledronic acid and IL-2 is more effective than their use in isolation. The already-established safety profiles of these agents make testing of the combination in GD2 positive cancers such as neuroblastoma or Ewing's sarcoma both rational and feasible.

## Abbreviations

FCS: Fetal calf serum
IPP: isopentenyl-5-pyrophosphate
TCR: T cell receptor
ADCC: antibody-dependent cellular cytotoxicity
mAb: monoclonal antibody
γδT cell: gamma delta T cell.

## Introduction

γδT cells are T cells that share characteristics of the innate immune system, recognizing markers of cellular stress or altered-self in an MHC-independent manner.^1,2^ This makes them potentially potent mediators of antitumor immunotherapy. However, they comprise only a small percentage of circulating T cells, and many tumors have developed means of evading γδT cell-mediated killing. There are a number of subsets of γδT cells, defined by their Vγ and Vδ chain usage, of which the most common in human blood expresses a relatively invariant Vγ9Vδ2 TCR. Vγ9Vδ2+ γδT cells have previously been shown to be potent killers of a range of hematological and solid tumor cell lines^3-5^ but in the majority of cases their cytotoxicity is significantly enhanced by target opsonization; and in diseases such as neuroblastoma is almost entirely antibody dependent,^5,6^ which is in part a reflection of that cancer's immunoinhibitory environment. For example, neuroblastoma cells secrete immunosuppressive factors such as soluble ligands of NKG2D, down regulate HLA-class I and produce immunosuppressive cytokines.^7–9^ It is unknown whether neuroblastoma cells are deficient in production of Vγ9Vδ2 γδTCR ligands such as phosphoantigens. From a cellular therapy perspective however, Vγ9Vδ2+ γδT cells are the most immediately feasible subset on account of the relative ease of expanding their numbers *in vivo* using the combination of zoledronate and IL-2, about which there is pre-existing safety data.^6^ There is some evidence of clinical efficacy in hematological and solid malignancy^10^ but results have been variable suggesting that additional combination treatments are required fully to harness the antitumor potential of γδT cells.

Neuroblastoma strongly expresses GD2, a ganglioside antigen, which is only very sparsely expressed on healthy tissue. Gangliosides are molecules composed of glycosphingolipids associated with one or more sialic acid residues. A number of monoclonal antibodies targeting GD2 are already in clinical use with promising results,^11–13^ although the mechanism underlying their action has not been fully elucidated. Immunotherapy using GD2-targeting antibodies has become a component of standard of care, first line treatment for high risk neuroblastoma, identifying this cancer type as an attractive model for development of further GD2-targeting immunotherapies. GD2 has been found at varying levels of expression on a number of other tumor types including Ewing's sarcoma,^14^ small cell lung cancer,^15^ osteosarcoma^16^ and melanoma^17^ suggesting that GD2-targeted immunotherapy should be further explored outside the neuroblastoma field. Indeed, its favorable differential expression has led to GD2 being ranked 12th in the National Cancer Institute list of most promising cancer antigens.^18^

Many immunotherapies that have been evaluated in clinical trials involve combinations of modalities. For example, in neuroblastoma the combination of cytokines (IL-2^+/–^ GM-CSF) with anti-GD2 monoclonal antibodies has been evaluated clinically.^11–13^ Researchers exploiting γδT cell-based immunotherapy have adopted two broad strategies; either stimulating a patient's γδT cells *in vivo* using systemic administration of zoledronate and IL-2, or using these agents for *ex vivo* expansion and adoptive transfer. Given the evidence that the cytotoxicity of Vγ9Vδ2+ γδT cells is significantly enhanced by target opsonization, there is a rationale for determining the efficacy of therapeutic combinations of lytic antibodies with agents to activate and expand γδT cells.^19^ Ch14.18 is a therapeutic anti-GD2 antibody currently in evaluation in a number of clinical trials, and thought to function predominantly by ADCC. It has not been extensively evaluated for killing function in combination with zoledronate and IL-2 in a range of cancer types expressing GD2. Here, we demonstrate that the combination of Vγ9Vδ2+ γδT cells, zoledronate and ch14.18 produced in CHO cells (ch14.18/CHO) leads to significant reductions in tumor growth compared to γδT cells and zoledronate alone, in two GD2-expressing disease models.

## Results

### Vδ1+ and Vδ1–/Vδ2– γδT cells kill Ewing's sarcoma cell lines in an antibody-independent manner

**Figure 1.**
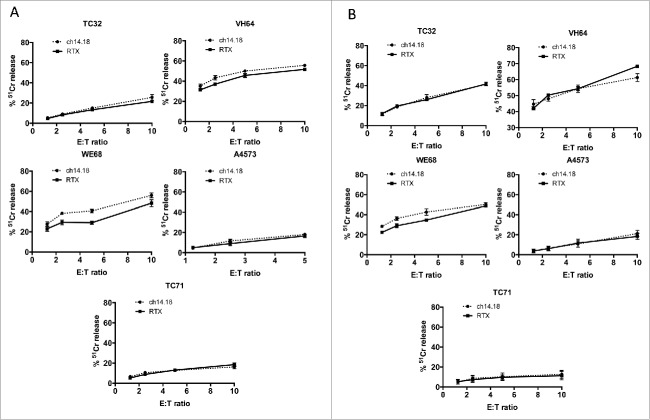
Killing of Ewing's sarcoma cell lines in 4 h chromium release assays by (**A**) Vδ1+ γδT cells and (**B**) Vδ1–/Vγ2– γδT cells. Representative data showing one of five donors.

**Ewing's** sarcoma has been reported as expressing GD2, making it a possible target for GD2-directed immunotherapy. We first evaluated the cytotoxic properties, against Ewing's cells, of γδT cells expanded using anti-γδTCR coated artificial antigen presenting cells as we have previously described.^5^ Vδ1^+^ (**Fig. 1A**) and Vδ1^−^/Vδ2^−^ (**Fig. 1B**) γδT cells killed a range of Ewing's sarcoma cell lines with varying levels of potency (range of killing of lines at 10:1 ET ratio of 15% to 55%, figures represent one of five representative donors). The addition of GD2-opsonizing antibody ch14.18/CHO made no significant difference to the level of cytotoxicity against any of the Ewing's sarcoma cell lines tested. This is consistent with our previous findings against neuroblastoma^5^ which indicate that Vδ1+ and Vδ1–/Vδ2– γδT cell cytotoxicity is antibody independent.

### GD2 on Ewing's sarcoma is an attractive target for Vγ9Vδ2+ γδT cell-mediated ADCC

**Figure 2.**
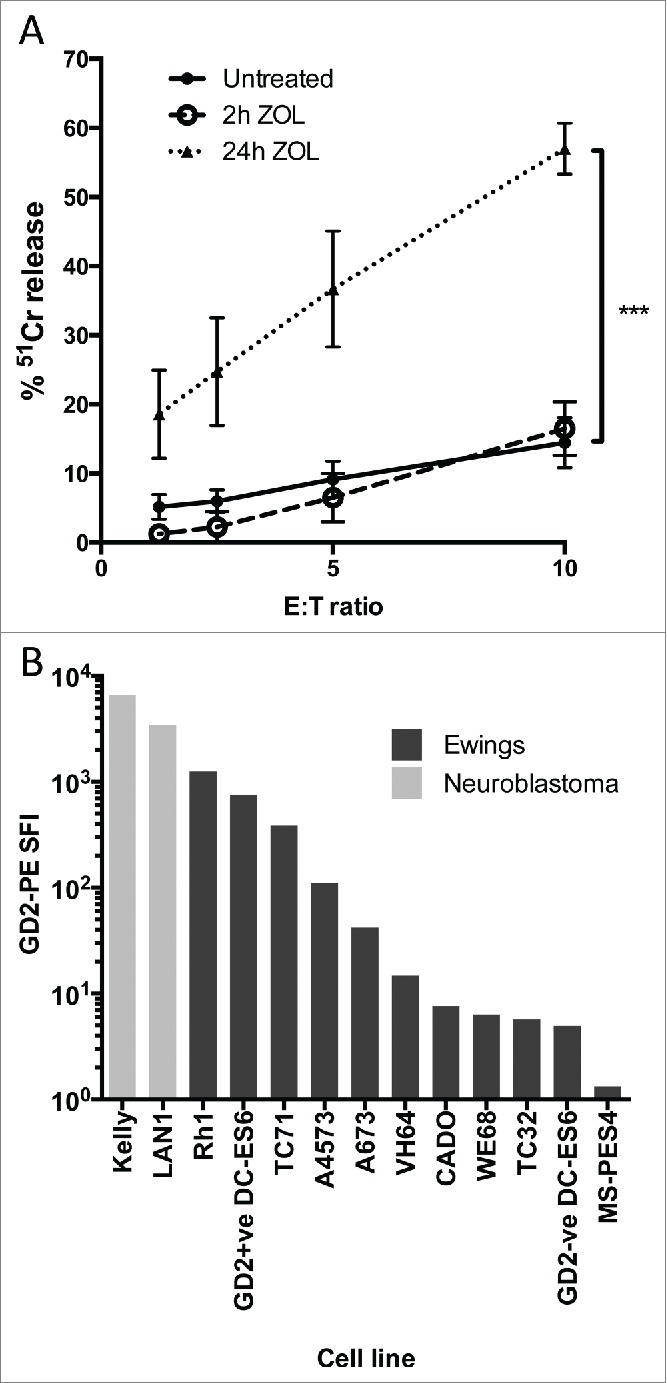
(**A**) 24 h treatment with 5 μM zoledronic acid sensitizes the Ewing's sarcoma cell line TC71 to antibody independent lysis by Vδ2+ γδT cells (*n* = 7, ***p = 0.001). (**B**) Comparative GD2 staining of Ewing's sarcoma and Neuroblastoma cell lines determined by flow cytometry and expressed as specific fluorescence intensity (SFI). SFI is calculated by dividing the median fluorescence intensity of a sample stained with PE-anti-GD2 with the median fluorescence intensity of a sample of the same cells stained with PE-isotype control.

There is currently no established method for specifically expanding Vδ1+ or Vδ1–/Vδ2– γδT cells *in vivo*, whereas Vγ9Vδ2+ γδT cell numbers can be increased both in blood and *ex vivo* using the combination of zoledronate and IL-2. Addition of zoledronate to target cells can also increase expression of Vγ9Vδ2+ TCR ligands, and thereby potentially sensitize them to Vγ9Vδ2 TCR-dependent killing. We first evaluated the effect of addition of 5 μM zoledronate to the Ewing's sarcoma line TC71, and observed a marked increase in sensitivity to killing by zoledronate-expanded Vγ9Vδ2+ γδT cells, only after a prolonged 24 h exposure (**Fig. 2A**, *n* = 7). However, after a standard intravenous dose, zoledronate has a peak plasma concentration of 1.13 μM, falling to <1% of this within 24 h^20^ suggesting the *in vitro* effect might not be translatable into clinical relevance. We therefore investigated whether combination treatment with antibody might be a more clinically relevant use of zoledronate-activated γδT cells to kill Ewing's sarcoma. GD2 was found to be heterogeneous for expression on Ewing's cell lines as judged by surface staining and flow cytometric analysis. While lower than that seen in the established GD2^bright^ neuroblastoma lines Kelly and LAN1 (**Fig. 2B** and **Fig. S1**), 9/10 Ewing's sarcoma lines had detectable expression of GD2 compared with isotype control staining. This is in agreement with other published data^14,21^ regarding GD2 expression in Ewing's sarcoma, and indicates that the antibody-dependent killing potential of Vδ2+ γδT cells could be exploited in this context. We generated isogenic GD2^bright^ clones of the naturally GD2^dim^ Ewing's sarcoma cell line DC-ES6 to allow us to determine antigen specific effects without confounding factors such as varying expression of other γδT cell stimulating ligands. GD2 expression in these engineered DC-ES6-GD2 cells was almost as bright as that seen on neuroblastoma lines LAN1 and Kelly (**Fig. 2B**).

### Vδ2+ γδT cells exhibit cytotoxicity against Ewing's sarcoma cell lines

**Figure 3.**
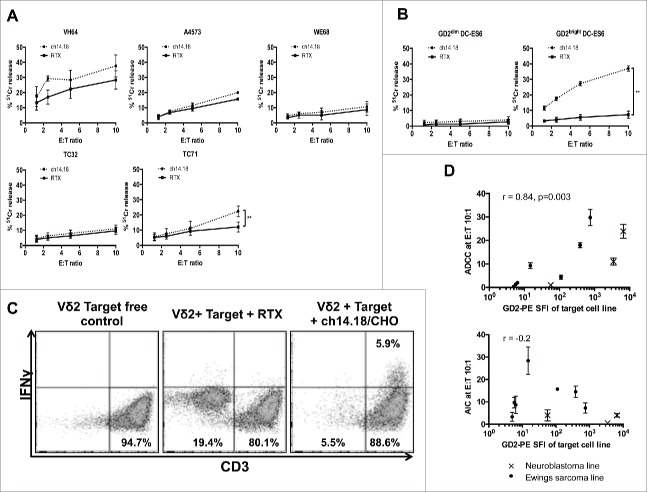
(**A**) Killing of wild-type Ewing's sarcoma cell lines by expanded Vδ2+ γδT cells opsonized with ch14.18/CHO anti-GD2 antibody or in the presence of a control antibody (Rituximab) (*n* = 3–6, **p = 0.0099). (**B**) Killing of isogenic GD2^bright^ and GD2^dim^ DC-ES6 Ewing's cell lines by Vδ2+ γδT cells in the presence of ch14.18/CHO anti-GD2 opsonizing antibody or Rituximab control antibody (*n* = 3, ***p = 0.0033). (**C**) IFNγ expression of Vδ2+ γδT cells in the presence of GD2^bright^ DC-ES6. Elimination of the DC-ES6 population and production of IFNγ is only seen when DC-ES6 is opsonized with ch14.18/CHO. (**D**) Correlation between the GD2 stain (PE-SFI) of Ewing's sarcoma and neuroblastoma cell lines and the degree to which Vδ2+ γδT cells exert ADCC or AIC against them at effector:target ratio 10:1. R value calculated by Spearman correlation.

We next evaluated ch14.18/CHO antibody-dependent and -independent killing of Ewing's lines by Vδ2+ γδT cells expanded using zoledronate or isopentenyl-5-pyrophosphate + IL-2. The antibody-independent killing in a 4 h cytotoxicity assay was in the range of 10–20% at E:T ratio of 10:1 (**Fig. 3A**, *n* = 3–6). Augmentation of cytotoxicity by target opsonization with ch14.18 was only observed in the GD2^bright^ cell line TC71 (*p* = 0.006, *n* = 6). Some enhancement of cytotoxicity was seen against Ewing's sarcoma cell lines expressing lower amounts of GD2 but this was not significant. Hence, there appeared to be a threshold level of GD2 below which ADCC was not observed. To confirm that this observation was due to the level of GD2 expression rather than other differences between cell lines, we compared killing of isogenic GD2^bright^ DC-ES6 with wild type GD2^dim^ DC-ES6 cells. Opsonization of GD2^bright^ DC-ES6 significantly enhanced killing by Vδ2+ γδT cells, which was minimal in the absence of ch14.18/CHO antibody (**Fig. 3B**). When Vδ2+ γδT cells were co-cultured overnight with GD2^bright^ DC-ES6, the target cells were eliminated and IFNγ was produced only when ch14.18/CHO was used as an opsonizing antibody (**Fig. 3C**, representative of three donors). To determine the relationship between antigen expression and the efficiency of antibody-dependent killing, we calculated how much cytotoxicity was antibody-dependent (killing with ch14.18/CHO minus killing with Rituximab control antibody) at a consistent effector:target ratio (10:1) in the panel of Ewing's lines and in Kelly and LAN-1 neuroblastoma lines. There was a strong correlation between antigen expression and ADCC (*r* = 0.87 by Spearman correlation, *p* = 0.002) but there was no correlation between GD2 expression and antibody-independent killing (**Fig. 3D**).

### Ch14.18/CHO alone does not directly kill Ewing's sarcoma even at supra-therapeutic concentrations

Published studies have demonstrated that binding of anti-GD2 antibodies to GD2^+^ neuroblastoma, melanoma and small cell lung cancer cell lines leads to increased levels of cell death via caspase-dependent and -independent pathways.^15,17,22^ This effect may increase the clinical efficacy of anti-GD2 antibodies in a cell-independent manner. We sought to determine whether this was the case against a panel of Ewing's cell lines; neuroblastoma cell lines are sensitive to this direct cytotoxicity^17^ and were therefore used as a positive control.

**Figure 4.**
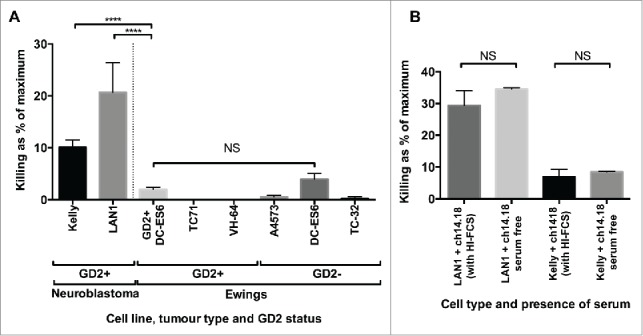
(**A**) To determine whether the correlation between ADCC and antigen expression was due to cellular stress imparted by the binding of antibody to GD2, we examined the killing of neuroblastoma and Ewing's sarcoma lines opsonized with ch14.18/CHO (20 μg/mL). High levels of spontaneous cell death were only seen in the GD2^+^ neuroblastoma lines, despite GD2 expression being higher in some of the Ewing's sarcoma lines (*n* = 9–42, depending on cell line, ****p = <0.0001). (**B**) To confirm that killing of neuroblastoma cell lines was not simply due to the binding and activation of residual complement in the serum, we compared the effects in the presence or absence of serum, and saw no significant difference (*n* = 5). The data is expressed as the “% of maximum killing” and represents the ^51^Cr release from opsonized cells incubated in medium for 4 h as a percentage of the ^51^Cr release of the same cells treated with Triton X, minus the paired result from cells treated with control antibody.

20 μg/mL of ch14.18/CHO was loaded onto a panel of GD2-expressing or non-expressing Ewing's sarcoma cell lines. The level of cell death after 4 h in the absence of immune effector cells was assessed using a ^51^Cr release assay and was compared to the maximum potential ^51^Cr release (obtained using Triton-X treatment) or the ^51^Cr release following coating with control antibody – Rituximab (anti-CD20 mAb) at identical concentration. The amount of cell death due to ch14.18 was determined by subtracting the spontaneous cell death in the Rituximab-treated controls from the cell death in ch14.18/CHO treated cells. There was detectable and reproducible direct cytotoxicity toward GD2 positive neuroblastoma cell lines Kelly and LAN1 by ch14.18/CHO. This effect was not seen in Ewing's sarcoma; ch14.18/CHO had minimal cytotoxicity at the same concentration against Ewing's sarcoma irrespective of GD2 expression; the difference between the killing of Kelly or LAN1 and GD2^bright^ DC-ES6, was highly significant (*p* =<0.0001). Furthermore, there was no significant difference in direct killing of the paired GD2^bright^ or GD2^dim^ DC-ES6 lines by ch14.18/CHO (**Fig. 4A**). To confirm that the killing of neuroblastoma cell lines was not the result of residual complement in the heat-inactivated fetal calf serum used in culture medium, we repeated the experiment after extensive washing and in the absence of serum. The presence or absence of serum had no significant influence, indicating that the killing of neuroblastoma was a direct effect of the antibody rather than a complement-mediated phenomenon (**Fig. 4B**).

### The combination of VγVδ2+ γδT cells, zoledronate and ch14.18/CHO impairs growth of established neuroblastoma and Ewing's sarcoma in a murine model

**Figure 5.**
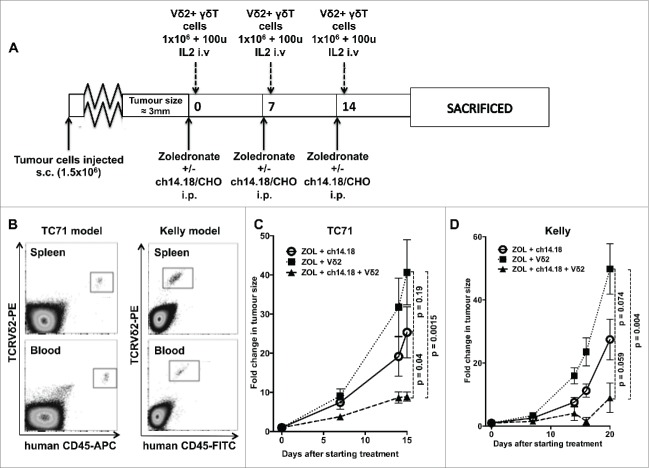
(**A**) Treatment schedule of mice used in *in vivo* experiments (**B**) Engraftment of Vγ9Vδ2+ γδT cells in NSG mice bearing TC71 Ewing's sarcoma (representative of six mice sampled) or Kelly neuroblastoma (representative of eight mice sampled) xenografts. Samples taken after culling, following three serial intravenous injections of 1 × 10^6^ γδT cells. (**C**) Fold change in tumor size of TC71 xenografts in NSG mice receiving Vγ9Vδ2 + zoledronate, Vγ9Vδ2 + zoledronate + ch14.18/CHO or zoledronate + ch14.18/CHO (**D**) – fold change in tumor size of Kelly xenografts in NSG mice receiving Vγ9Vδ2 + zoledronate, Vγ9Vδ2 + zoledronate + ch14.18/CHO or zoledronate + ch14.18/CHO.

Because of fundamental differences between murine and human γδT cells, *in vivo* modeling of γδT cell function in mice requires the use of immunodeficient animals. We evaluated the effect of adoptive transfer of human zoledronate expanded γδT cells on subcutaneous xenografts of GD2-expressing Ewing's sarcoma cells (TC71) in NOD.Cg-*Prkdc*^*scid*^ *Il2rg*^*tm1Wjl*^/SzJ (NSG) mice which have no endogenous T or NK cells. Our animal model of neuroblastoma used subcutaneous xenografts of the GD2-expressing cell line Kelly, which we have shown to be susceptible to Vγ9Vδ2+ γδT cell-mediated ADCC *in vitro*.^5^ We chose TC71 as an un-manipulated GD2-expressing model of Ewing's sarcoma for comparison. Mice with small established tumors were randomly assigned to treatment groups, this design ensuring that therapeutic benefits of intravenous injections of Vγ9Vδ2+ γδT cells relies not only on the γδT cells being able to kill the tumor cells, but also on their ability to infiltrate the tumor from the bloodstream. Weekly injections of zoledronate (120 μg/kg) +/– ch14.18/CHO (300 μg per mouse) and relatively low doses of Vγ9Vδ2+ γδT cells (1 × 10^6^ cells per week) were given alongside low-dose IL-2 (100 u/mouse i.v.) (**Fig. 5A**) and engraftment was evaluated by flow cytometric analysis of blood and spleen taken at the end of the experiment (following three cycles of treatment), confirming successful engraftment of transferred cells in all mice sampled (**Fig. 5B**). In the absence of zoledronate, γδT cells failed to engraft, even when much higher doses (1 × 10^7^ cells per dose) were used. Following three cycles of combination treatment with zoledronate, ch14.18/CHO and Vγ9Vδ2+ γδT cells, tumor growth was significantly impaired when compared to γδT cells + zoledronate only (*p* = 0.0015) or with ch14.18/CHO + zoledronate (*p* = 0.04) (**Fig. 5C**, *n* = 4–6 per group). Interestingly, there was a suggestion of some inhibition of growth from the combination of ch14.18/CHO and zoledronate alone in the absence of any human γδT cells though this was not significantly different from the tumor growth rate seen with γδT cells + zoledronate only (*p* = 0.19).

In the neuroblastoma model, the same pattern was observed. In Vγ9Vδ2 + zoledronate treated mice, there was exponential tumor growth; however, when ch14.18/CHO was administered alongside the zoledronate and Vγ9Vδ2^+^ cells, there was a significant reduction in growth with tumor shrinkage in 2/4 mice. By 20 d following the commencement of treatment (after three cycles of treatment), the difference in growth rate was highly significant between mice receiving zoledronate + Vγ9Vδ2 and those receiving zoledronate + Vγ9Vδ2 + ch14.18/CHO (*p* = 0.004, *n* = 4, **Fig. 5D**). As was observed in the Ewing's model, the addition of ch14.18/CHO + zoledronate in the absence of Vγ9Vδ2+ γδT cells also led to a non-significant reduction in tumor growth compared with the Vδ2 + zoledronate treatment. This minor effect of ch14.18/CHO + zoledronate was consistently observed in repeated experiments in both the Ewing's and neuroblastoma models.

## Discussion

We show here that the cytolytic properties of Vγ9Vδ2 γδT cells are preserved in an *in vivo* setting, making these findings highly translatable to early phase trials. Our new data, combined with results that we have previously published in neuroblastoma^5^ indicate that maximization of this cytolytic activity has a number of prerequisites – γδTCR engagement and engagement of ADCC via CD16.

Many transformed cells produce increased quantities of mavalonate pathway intermediates such as isopentenyl-5-pyrophosphaste (IPP) which lead to engagement of the Vγ9Vδ2 TCR and stimulate the γδT cells – so called “signal 1.” γδTCR stimulation alone is insufficient to fully activate Vγ9Vδ2+ γδT cells; an additional co-stimulatory signal via receptors such as NKG2D is also required.^23^ Transformed cells, including Ewing's sarcoma,^24^ typically express membrane bound NKG2D ligands facilitating this costimulation and sensitizing them to cytotoxic killing; however, some tumors including neuroblastoma have been demonstrated to shed soluble NKG2D ligands which can block NKG2D cross-linking and therefore abrogate this essential second signal.^9^ This phenomenon may in part explain the low levels of antibody-independent killing exerted by Vγ9Vδ2+ γδT cells against neuroblastoma. Unlike αβT cells, Vδ2+ γδT cells express high levels of the low-affinity Fc Receptor FcγRIII (CD16), the functional role of which in ADCC is most evident in NK cells and macrophages. We previously showed that γδT express only very low levels of the high-affinity Fcγ receptors CD32 and CD64 indicating that ADCC induced by IgG antibodies occurs via CD16 ligation. CD16 provides an alternative route for Vγ9Vδ2+ γδT cell co-stimulation in the presence of an appropriately opsonized target. Naturally, there is variation in the balance of γδTCR and co-stimulatory stimulus between tumor types and also between cell lines of similar tumor origin. This variability presents problems for the therapeutic use of Vγ9Vδ2+ γδT cells as it provides additional avenues of immune escape.

To overcome this variability, the stimulus to either the γδTCR, co-stimulatory receptors or both can be artificially enhanced. Treatment of target cells with zoledronate increases IPP production and thereby increases the availability of γδTCR stimulus,^25^ sensitizing tumor cells to Vγ9Vδ2+ γδT cell-mediated killing.^26,27^ The evidence for the clinical efficacy of zoledronate + IL-2 combination treatment in cancer clinical trials is however limited,^6^ suggesting that approaches for further strengthening of the γδT cell response must be explored.

In NK cells, CD16 engages CD3ζ or FcεRIγ homo/heterodimers, which bear immunoreceptor tyrosine-based activation motifs (ITAMs), facilitating recruitment of tyrosine kinases ZAP70 and Syk. This triggers a signaling cascade involving SLP-76, the p85 subunit of PI3-kinase and Grb2 leading to an increase in intracellular Ca^2+^ levels. The breadth of signaling that occurs following CD16 ligation in NK cells is quite different from the relatively small subset of molecules which are implicated in the conventional T cell “signal 2” via co-stimulatory receptors such as CD28. CD28 signaling is viewed as both qualitative (producing a specific signal via a narrowly defined pathway) *and* quantitative (with effect determined by signal strength).^28^ Our data demonstrating the correlation of the effect (ADCC) with GD2 staining suggests that the functional effects of CD16 ligation in Vγ9Vδ2+ γδT cells are quantitative – the strength of the signal is directly proportional to the level of binding to the target cell in a context where epitopes are likely to be saturated with antibody. The level of *in vitro* antibody-dependent killing exerted by γδT cells has previously been shown to correlate with the FcγRIII (CD16) expression of the effectors in question.^5,29^ Our data support the hypothesis that the number of complete antigen-antibody-CD16 complexes is what influences the strength of the signal.

### Translational relevance of Vγ9Vδ2+ γδT cell ADCC

γδT cells are potent effectors of antibody-dependent cytotoxicity that can be expanded in a specific manner without bystander activation of other immune effectors.^30^ A number of clinical trials have already demonstrated that treating cancer patients with a combination of zoledronate and low dose IL-2 can lead to objective clinical responses in those patients where γδT cell expansion is achieved.^6,30–32^ Target opsonization with appropriate antibody led to significant increases in Vγ9Vδ2^+^ cytotoxicity against tumor cells in a number of previous *in vitro* studies^3,5,19,29,33^ but there is a paucity of *in vivo* evidence supporting the combination of Vγ9Vδ2+ γδT cell activation and tumor-targeting antibody,^19,33^ and this approach has never been attempted in a clinical setting despite the availability of appropriate agents with known safety profiles in adults and children.

Neuroblastoma is a potential model for this kind of combination treatment. GD2 expression in neuroblastoma is preserved following treatment with anti-GD2 monoclonal antibodies, avoiding problems of antigen loss which are often seen in other antibody-targeted malignancies such as CD20 expressing lymphoma. This makes early phase trials in patients with refractory or relapsed GD2-expressing disease a much more feasible prospect. Here, we have demonstrated that, in the context of neuroblastoma or Ewing's sarcoma expressing high levels of GD2, Vγ9Vδ2+ γδT cells can exert an antibody-dependent suppression of tumor growth, even at low doses. While the clinical utility of targeting GD2 on neuroblastoma has already been validated, it remains to be seen whether this will be of benefit in Ewing's sarcoma due to the heterogeneity of GD2 expression seen in this disease.

The ability of Vγ9Vδ2+ γδT cells to exert *in vivo* ADCC against solid tumors is highly translatable and may have relevance in the context of other tumor associated antigens against which there are therapeutic antibodies. Furthermore, as there is already extensive phase I safety data on the combination of zoledronate + IL-2 for *in vivo* γδT cell expansion^4,10,30-32^ and also on the combination of anti-GD2 antibodies + IL-2,^11,12^ combining these two approaches would offer a route to studying the benefits of providing an antibody alongside a specifically expanded ADCC competent effector population.

## Materials and Methods

### Statistical analysis

Statistical analyses were performed using GraphPad Prism Version 6.0e. Error bars, where displayed, indicate the standard error of the mean of data from replicate independent experiments. Significance of difference between samples within figures was confirmed using paired or unpaired *t*-tests, depending on the experimental setting, with *p* = <0.05 indicating significance. Correlation between variables was demonstrated by Spearman rank correlation coefficient.

### Cell lines

Human neuroblastoma cells lines Kelly and LAN1 and the human Ewing's sarcoma cell lines A673 and Rh1 were originally obtained from the ATCC. The Ewing's sarcoma cell lines TC-71 and Cado were from DSMZ (Braunschweig, Germany). VH-64 and WE-68 cells were gifts from Frans van Valen's laboratory at the Institute of Experimental Orthopedics of University of Muenster, Germany. A-4573 and TC-32 were from the cell line bank at Children's Hospital Los Angeles. MS-PES4 and DC-ES6 were established by our group as described previously.^14^

### Expansion of γδT cells from PBMC

γδT cells were expanded from freshly isolated peripheral blood mononuclear cells (PBMCs) or from isolated pure populations of γδT cells. Cells were obtained from healthy donors following institutional review board approval. Zoledronate-based expansions of Vγ9Vδ2+ γδT cells were performed by culturing freshly isolated PBMC in RPMI-1640 medium containing 10% Fetal calf serum, 1% Penicillin/Streptomycin, 5 μM zoledronate (Zometa – Novartis) and 100 IU/mL IL-2 (PeproTech 200–02), which was refreshed three times weekly. After 14 d of expansion, γδT cell isolation was performed using the Miltenyi γδT cell isolation kit (130-092-892) in accordance with the manufacturer's protocol. Expansion of Vδ1+ or Vδ1–/Vδ2– γδT cells were performed as previously described.^5^ Cells were incubated at 37°C, 5% CO2.

### Generation of GD2^+^ DC-ES6

Wild-type DC-ES6 were transduced with SFG gammaretrovirus encoding GD2 and GD3 synthase co-expressed via foot and mouth virus 2A self-cleaving peptide sequence, kindly provided by M Pule (UCL). After transduction, single GD2-expressing clones were generated by limiting dilution, and expanded.

### Antibodies

The following antibodies were used in this investigation: Treatment grade antibodies Ch14.18/CHO and rituximab were provided by Great Ormond Street Hospital pharmacy. Mouse anti-human CD3-PE/Cy7 (Biolegend 3000316; clone HIT3a), mouse anti-human TCR Vδ1-FITC (Thermo Scientific TCR2730; clone TS8.2), mouse anti-human TCR Vδ2-PE (BioLegend 331408; clone B6), mouse anti-human CD45-APC (Biolegend 304012; clone HI30), mouse anti-human CD45-APC (Biolegend 304006; clone HI30), rat anti-human/mouse CD11b (Biolegend 101228; clone M1/70), mouse anti-human IFNγ-APCCy7 (Biolegend 502530; clone 4S.B3). Live/dead staining was performed using Zombie^TM^ Yellow fixable viability kit (Biolegend 423104), mouse anti-ganglioside GD2-PE (Biolegend 357304, clone 14G2a). Compensation was carried out using single-color controls and eBioScience OneComp eBeads (eBioScience 01-1111). When experiments regarding comparative GD2 expression of neuroblastoma and Ewing's sarcoma cell lines were being carried out, 0.5 × 10^6^ cells were resuspended in 100 μL of buffer and labeled with 3μL of antibody (final concentration 1.5 μg/mL) for 10 min in the fridge before washing twice in PBS. Flow cytometry analysis was carried out on BD LSRII or BD FACSAria flow cytometers and results were analyzed using FlowJo vX.0.7.

### Cytotoxicity assays

Expanded and purified γδT cells were used as effector cells. Target cells were coated either with opsonizing antibody or a non-targeting isotype control and labeled with 100 μCi Na_2_^51^CrO_4_ in cell culture and tested in a standard chromium release assay as previously described. A range of E:T ratios was used in each case (10:1, 5:1, 2.5:1, 1.25:1) and cells were incubated for 4 h for each assay. Anti-GD2 antibody ch14.18/CHO (clinical grade) was used as a GD2-specific opsonizing antibody, with rituximab anti-CD20 (clinical grade) as a control.

The direct cytotoxicity of ch14.18/CHO was determined by the following formula (background 51Cr release from ch14.18/CHO opsonized/Triton-X induced 51Cr release from ch14.18/CHO opsonized) – (background 51Cr release from Rituximab treated cells/Triton-X-induced 51Cr release from Rituximab-treated cells).

### Animal experiments

All experiments and procedures involving animals were conducted in accordance with the relevant laws and institutional procedures, approved by the UK government. Mice were housed in pathogen-free conditions in individually ventilated cages. Animals were originally obtained from Charles River Laboratories (Wilmington, MA) and a breeding colony was subsequently generated and maintained locally.

Adult female NOD.Cg-*Prkdc*^*scid*^ *Il2rg*^*tm1Wjl*^/SzJ (NSG) mice were injected with 1.5 × 10^6^ tumor cells (TC-71 or Kelly) subcutaneously, and tumor growth was monitored using electronic callipers. Once the tumors reached approximately 3 mm in diameter, mice were randomized to treatment groups and groups were coded in a way that had no relation to the treatment received, to aid blinding. Weekly intraperitoneal injections of zoledronate (120 μg/kg) +/- ch14.18/CHO (300 μg per mouse) were administered. In mice who were assigned to the groups receiving γδT cells, 1 × 10^6^ purified Vδ2^+^ cells were injected intravenously on the day after injection of the zoledronate+ch14.18/CHO. 100 u of human IL-2 were injected intravenously alongside the γδT cells. Fold change in tumor size was calculated based on the size at Day 0 of treatment. Mice were culled when the average tumor diameter reached 15 mm. Blood and spleen samples were taken after culling. Blood samples were treated with ACK lysis buffer (Life Technologies A10492-01) before staining for FACS analysis. Spleen samples were passed through a 70 μM cell strainer before being treated with ACK lysis buffer and being stained for FACS analysis to confirm Vδ2+ γδT cell engraftment.

## Supplementary Material

supplemental_fig._1.pdf
