# Supplementary figures and images for: Effective combination treatment of GD2-expressing neuroblastoma and Ewing's sarcoma using anti-GD2 ch14.18/CHO antibody with Vγ9Vδ2+ γδT cells
Source: Oncoimmunology. 2015 Apr 27;5(1):e1025194. doi: 10.1080/2162402X.2015.1025194 (PMC4760299; doi:10.1080/2162402X.2015.1025194)

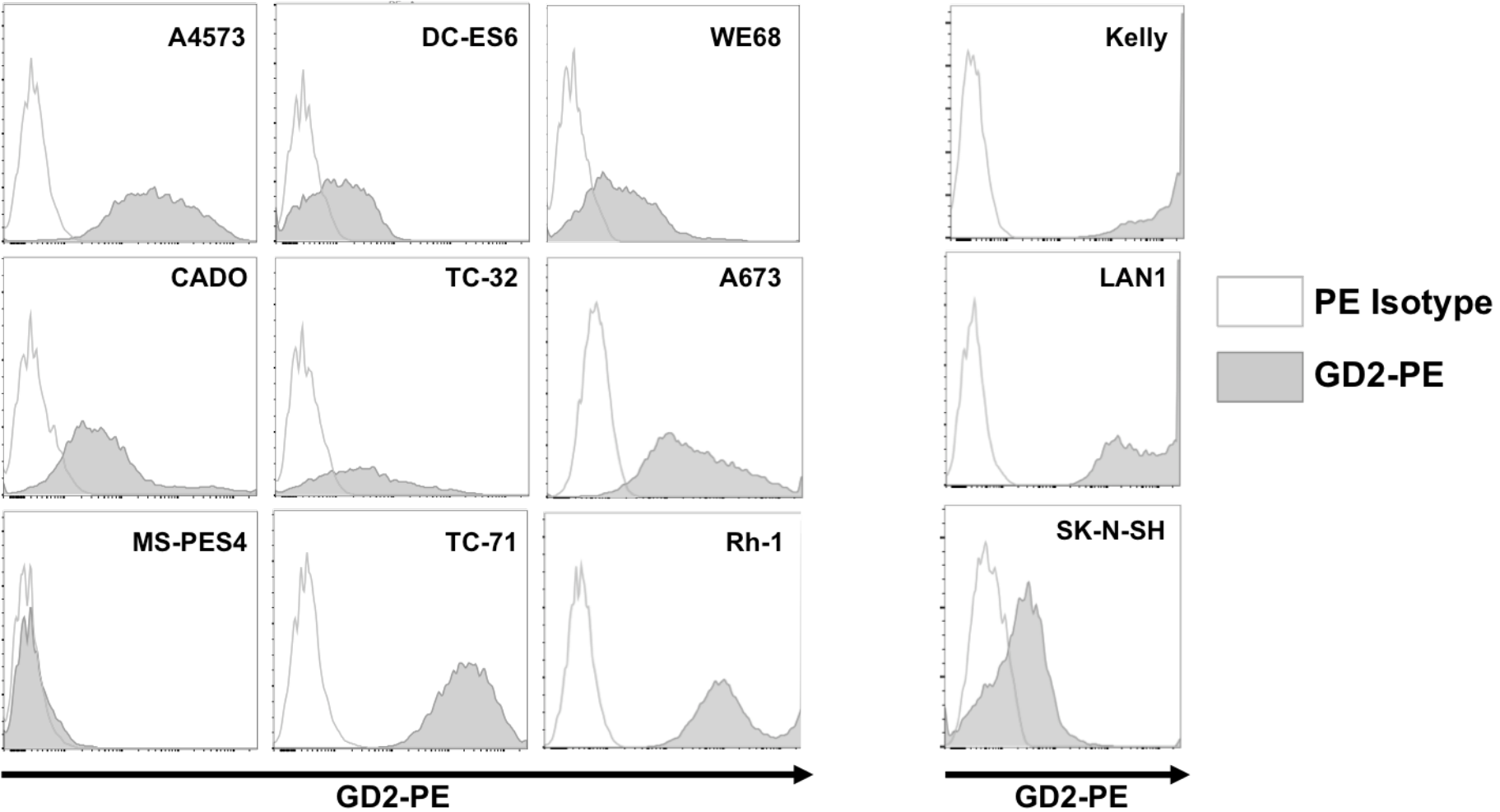

Supplement: supplemental_fig._1.pdf [file koni-05-01-1025194-s001.pdf]
